# Supplementary material for: Origins of life: first came evolutionary dynamics
Source: QRB Discov. 2023 Mar 22;4:e4. doi: 10.1017/qrd.2023.2 (PMC10392681; doi:10.1017/qrd.2023.2)
Supplement: Supplementary file 1 [file S2633289223000029sup001.pdf]

SI for Origins of life: First came evolutionary dynamics

Charles Kocher[1,2] and Ken A. Dill[1,2,3,*]

[1] *Laufer Center for Physical and Quantitative Biology, Stony Brook University*
[2] *Department of Physics and Astronomy, Stony Brook University*
[3] *Department of Chemistry, Stony Brook University*

When life arose from prebiotic molecules 3.5 billion years ago, what came first? Informational molecules (RNA, DNA), functional ones (proteins), or something else? We argue here for a different logic: rather than seeking a *molecule type,* we seek a *dynamical process.* Biology required an ability to evolve before it could choose and optimize materials. We hypothesize that the *evolution process* was rooted in the *peptide folding process.* Modeling shows how short random peptides can collapse in water, catalyze elongation of others, powering both increased folding stability and emergent autocatalysis through a disorder-to-order process.

SI.1: THE SECOND LAW IS NOT THE GOVERNING PRINCIPLE OF EVOLUTION

Evolution is governed by principles of transport, not equilibrium. Concepts of equilibrium thermodynamics are sometimes mistakenly regarded as the physical governing principle that arches over evolution and origins of life. For example, Erwin Schrodinger's famous book, "What is Life?" (Schrodinger 1944), sees biology's emergence as a battle against the entropy of the Second Law of Thermodynamics. And, it is often argued that biology's tendency toward "complexity" would violate Second-Law tendencies toward disorder (Morris 1974). Yet, as noted in the main text, equilibrium tendencies, such as expressed in the Second Law of Thermodynamics, are not the governing principle of biological evolution as a dynamical process. While equilibrium describes the limiting state of zero forces and gradients, nonequilibria are described instead by the forces and flows of transport phenomena. Fig S1 illustrates three realms – equilibrium (the limit of zero force), driven NEQ (subject to applied forces), and driven adaptive NEQ (where the system not only responds to applied force, but also changes its fundamental properties to respond differently in the future). Biological systems are both driven and adaptive.

Biological "complexity" is not the same as thermodynamic entropy or order. Biology evolves toward increased fitness, not toward increased complexity or decreased thermal entropy. Biological adaptations are best described as makers becoming better at making in the face of environmental conditions and their changes (Merindol and Walther 2017, Pascal and Pross 2015, Pross 2019).

SI.2: "SURVIVAL" OF THE "FITTEST"

One goal of modeling is to pin down concepts quantitatively. The idea that the force of evolution is "survival of the fittest" – which is a term that dates back to Herbert Spencer (Spencer 1864) and Charles Darwin (Darwin 1964) – raises some questions for quantitative modeling. First, if an environment is at steady-state, and if there is a single dominant evolutionary degree of freedom, then fitness landscapes are useful descriptors (Agozzino et al. 2020, De Visser and Krug 2014, Wright 1932). But defining fitness and survival can be more complicated if there are multiple coupled degrees of freedom, or if multiple species can survive at the same time. Then, there can be "dynamical aspects" – such as predators chasing prey – that fitness landscapes alone don't convey (Zhang et al. 2012). Indeed, the inadequacy of the Wrightian definition of fitness landscapes was discussed by Crow and Kimura in their population genetics textbook ("A note on terminology" at the end of Chapter 5 section 7, pg. 224-5 of the cited version) (Crow and Kimura 2009).

Second, even more challenging is when environments are themselves changing dynamically. In steady-state environments, competitive success can often be expressed as R^* = *death rate divided by eating rate* in Tilman ecological

---

* To whom correspondence should be addressed: dill@laufercenter.org

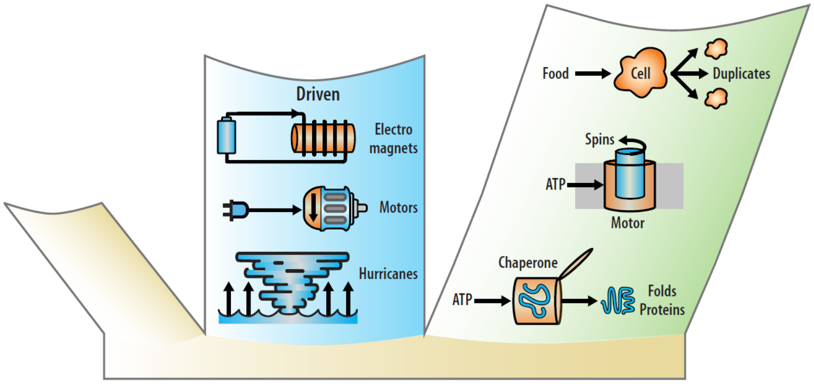

FIG. S1. **Three stovepipes of dynamics:** (Left, yellow) Relaxations and *Equilibria* (EQ). (Middle, blue) Nonequilibrium (NEQ) *driven* processes, such as electromagnets, motors, hurricanes. (Right, green) *Driven & adaptive* processes, such as those that occur in cells that evolve.

models (Hsu et al. 1977, Lobry et al. 2006, Tilman 1982, van Opheusden et al. 2015), a measure of relative populations of species. But, in non-steady-state environments, such as booms and busts of resources (day/night, seasonal, etc.), it's trickier to define competitive success. The relative populations become time-dependent, and Mom A who's ahead immediately after the bust may fall behind later. Moreover, even if Mom A is the winner in environment E_1, it's not predictive of which Mom will win in the next (unpredictable) environment E_2. For example, say Mom A can only survive during a boom period, but is not robust enough to survive at lower resource levels. And, is a Mom more successful if she has a higher population at time t or a lower population at t but is more persistent for longer times? Might a more useful metric be economists' notion of "present expectation of future integrated value," adapted to this situation?

Third, in *repeatably periodic* non-steady environments, some organisms can switch between multiple internal programs. Examples include sleep-wake cycles, or sporulation and hibernation, or switching to strongly reduced metabololism – in tardigrade "tun" formation (Soemme 1996, Wełnicz et al. 2011) or in microbes trapped in ocean sediments for tens of millions of years in oxygen-free environments (Morono et al. 2020).

Finally, while we have noted that fitness is a uniquely biological concept – entailing the many ways that cells and organisms can be self-serving – the origins of life and the origins of fitness require some precursor that was physico-chemical and molecular. How might molecules have become self-serving? We have suggested in the main text that the molecular precursor might simply have been *persistence*, the greater stability of some molecular states for longer times than other states, in environments that are either fixed or unruly.

SI.3: FURTHER STEPS TOWARD ORIGINS OF LIFE

We have advocated here for the crucial requirement, as a predicate before life can arise, of some autocatalytic dynamical mechanism that can propagate competitive advancement. We indicated how the most natural molecular vehicle for that dynamics is protein molecules. But, this alone is not sufficient to define the origin of life. We view minimal life as having the following: some form of DNA- or RNA-like memory, as both the keeper of fitness information and also linkage among generations; some form of cell-like encapsulation or droplet into unit individuals (the SELF) that are the carriers of lineages and controllers of inputs and outputs; some form of individualized onboard energy currency, such as the ATP; and effective functional biochemical networks. These components would give more persistence and fitness beyond DEM dynamics alone. While the DEM is necessary, it is not sufficient. Our present work makes no predictions about when and how these other components became incorporated, including the possibility they arose in parallel with the DEM. However, it is clear that once the DEM arose, its best moms discovering mechanisms for more faithful replication and more efficient energy usage would be natural ways to increase fitness and persistence.

The present work has some implications. First, it is possible the DEM could have arisen before other components because it propagates stably on its own. It would imply that the proteins being synthesized could all coexist in the same "warm pond" (or other) space. Prior to individuals and lineages, the DEM might have been delocalized, a sort of a communal pond that then funnels down to lineages. Such parallel coexistence has been suggested before

(Crick 1968, Dyson 1999, Vetsigian et al. 2006). Second, the present aligns with Dyson's view that metabolism could have preceded replication (Dyson 1999), and/or that proteins and RNAs interacted with each other (Carter Jr and Kraut 1974, Frenkel-Pinter et al. 2020), but does not align with the view that small-molecule reactions could precede mobile catalysts. The DEM is fundamentally a process that acts on molecule makers and catalysts. For that same reason, the DEM perspective does not align with the *amyloid hypothesis*, whereby the first protein molecules were essentially aggregates (Maury 2009, 2015, 2018), since protein aggregates don't tend to have specific sites of catalysis or functional actions or sequence $\rightarrow$ function properties. In addition, there is a view, called "the selfish gene" (Dawkins 1978), that DNA and genes are the drivers of their own evolution. The DEM perspective is that evolution is driven foremost by functionality and molecule-making, and the informational role could arise later. In fact, these two views can be reconciled. Instead of viewing DNA and genes as the driver (DNA uses proteins to make more DNA), it is equivalent to view proteins as the driver (proteins use DNA to make more proteins). The interpretation, then, is that the Darwinian evolution arose in proteins/maker molecules and has continued acting on them ever since, a point made stronger by noting that selective pressures in biological evolution do not act on genotype (DNA) directly, but instead on phenotype (proteins). Genetic coding of amino acids could have come about through aminoacyl-tRNA synthetase duality (Carter Jr and Wills 2021).

We should point out here as well that the foldcat idea could extend to ribozymes. There is a natural mechanism (complementary base pair bonding) for a "foldcat" RNA to attach to a client chain and a free monomer to spatially localize them for ligation. The foldcat mechanism, or in this case a type of templated polymerization, could have played a part in the emergence of both nucleic acids and proteins. For nucleic acids, the foldcat mechanism has the added benefit of creating a complementary RNA strand; some sequence information is preserved. However, for the reasons we mentioned in the main text on why we focus on proteins, the protein-like foldcat mechanism would be expected to be more potent than a nucleic acid foldcat mechanism.

Finally, we note two relevant works here. First, in a soup of proteins, how might those proteins have become chained together into functional biochemical pathways? A catalyst chemotaxis mechanism has been explored in computer simulations of the *Producer Recruitment Model*, which shows how functional molecules can diffuse together and associate if they have a substrate or product in common (Kocher et al. 2021). It is a reversal of the well-known paradigm of *structure dictates function*, whereby *function dictates structure.* Second, if a DEM process is producing diverse proteins, and if it occurs in proto-cells with random RNA molecules, a computer simulation of *the Bootstrap Model* shows how the two polymer types can come together to form fruitful associations and networks (Farquharson et al. 2022).

Agozzino, L., Balázsi, G., Wang, J., and Dill, K. A. (2020). How do cells adapt? stories told in landscapes. *Annual Review of Chemical and Biomolecular Engineering*, 11:155–182.

Carter Jr, C. W. and Kraut, J. (1974). A proposed model for interaction of polypeptides with rna. *Proceedings of the National Academy of Sciences*, 71(2):283–287.

Carter Jr, C. W. and Wills, P. R. (2021). The roots of genetic coding in aminoacyl-trna synthetase duality. *Annual review of biochemistry*, 90:349–373.

Crick, F. H. (1968). The origin of the genetic code. *Journal of molecular biology*, 38(3):367–379.

Crow, J. and Kimura, M. (2009). *An Introduction to Population Genetics Theory.* Blackburn Press.

Darwin, C. (1964). *On the origin of species: A facsimile of the first edition.* Harvard University Press.

Dawkins, R. (1978). *The Selfish Gene.* Paladin: Science. Paladin, Granada Publishing Limited.

De Visser, J. and Krug, J. (2014). Empirical fitness landscapes and the predictability of evolution. *Nature Reviews Genetics*, 15(7):480–490.

Dyson, F. (1999). *Origins of life.* Cambridge University Press.

Farquharson, T., Agozzino, L., and Dill, K. (2022). The bootstrap model of prebiotic networks of proteins and nucleic acids. *Life*, 12(5):724.

Frenkel-Pinter, M., Haynes, J. W., Mohyeldin, A. M., Sargon, A. B., Petrov, A. S., Krishnamurthy, R., Hud, N. V., Williams, L. D., and Leman, L. J. (2020). Mutually stabilizing interactions between proto-peptides and rna. *Nature Communications*, 11(1):3137.

Hsu, S.-B., Hubbell, S., and Waltman, P. (1977). A mathematical theory for single-nutrient competition in continuous cultures of micro-organisms. *SIAM Journal on Applied Mathematics*, 32(2):366–383.

Kocher, C., Agozzino, L., and Dill, K. (2021). Nanoscale catalyst chemotaxis can drive the assembly of functional pathways. *The Journal of Physical Chemistry B*, 125(31):8781–8786.

Lobry, C., Rapaport, A., and Mazenc, F. (2006). Sur un modèle densité-dépendant de compétition pour une ressource. *Comptes Rendus Biologies*, 329(2):63–70.

Maury, C. P. J. (2009). Self-propagating β-sheet polypeptide structures as prebiotic informational molecular entities: the amyloid world. *Origins of Life and Evolution of Biospheres*, 39(2):141–150.

Maury, C. P. J. (2015). Origin of life. primordial genetics: Information transfer in a pre-rna world based on self-replicating beta-sheet amyloid conformers. *Journal of Theoretical Biology*, 382:292–297.

Maury, C. P. J. (2018). Amyloid and the origin of life: self-replicating catalytic amyloids as prebiotic informational and protometabolic entities. *Cellular and Molecular Life Sciences*, 75(9):1499–1507.

Merindol, R. and Walther, A. (2017). Materials learning from life: concepts for active, adaptive and autonomous molecular systems. *Chemical Society Reviews*, 46(18):5588–5619.

Morono, Y., Ito, M., Hoshino, T., Terada, T., Hori, T., Ikehara, M., D'Hondt, S., and Inagaki, F. (2020). Aerobic microbial life persists in oxic marine sediment as old as 101.5 million years. *Nature communications*, 11(1):1–9.

Morris, H. M. (1974). *Scientific Creationism (Henry Morris Signature Collection).* New Leaf Publishing Group.

Pascal, R. and Pross, A. (2015). Stability and its manifestation in the chemical and biological worlds. *Chemical Communications*, 51(90):16160–16165.

Pross, A. (2019). Seeking to uncover biology's chemical roots. *Emerging Topics in Life Sciences*, 3(5):435–443.

Schrodinger, E. (1944). *What is life?* Cambridge University Press.

Soemme, L. (1996). Anhydrobiosis and cold tolerance in tardigrades. *European Journal of Entomology*, 93:349–358.

Spencer, H. (1864). *The Principles of Biology.* Number v. 1 in A system of synthetic philosophy. Williams and Norgate.

Tilman, D. (1982). *Resource Competition and Community Structure.* Monographs in Population Biology. Princeton University Press.

van Opheusden, J. H., Hemerik, L., van Opheusden, M., and van der Werf, W. (2015). Competition for resources: complicated dynamics in the simple tilman model. *SpringerPlus*, 4(1):1–31.

Vetsigian, K., Woese, C., and Goldenfeld, N. (2006). Collective evolution and the genetic code. *Proceedings of the National Academy of Sciences*, 103(28):10696–10701.

Wełnicz, W., Grohme, M. A., Kaczmarek, Ł., Schill, R. O., and Frohme, M. (2011). Anhydrobiosis in tardigrades—the last decade. *Journal of insect physiology*, 57(5):577–583.

Wright, S. (1932). *The roles of mutation, inbreeding, crossbreeding, and selection in evolution*, volume 1. na.

Zhang, F., Xu, L., Zhang, K., Wang, E., and Wang, J. (2012). The potential and flux landscape theory of evolution. *The Journal of chemical physics*, 137(6):065102.
